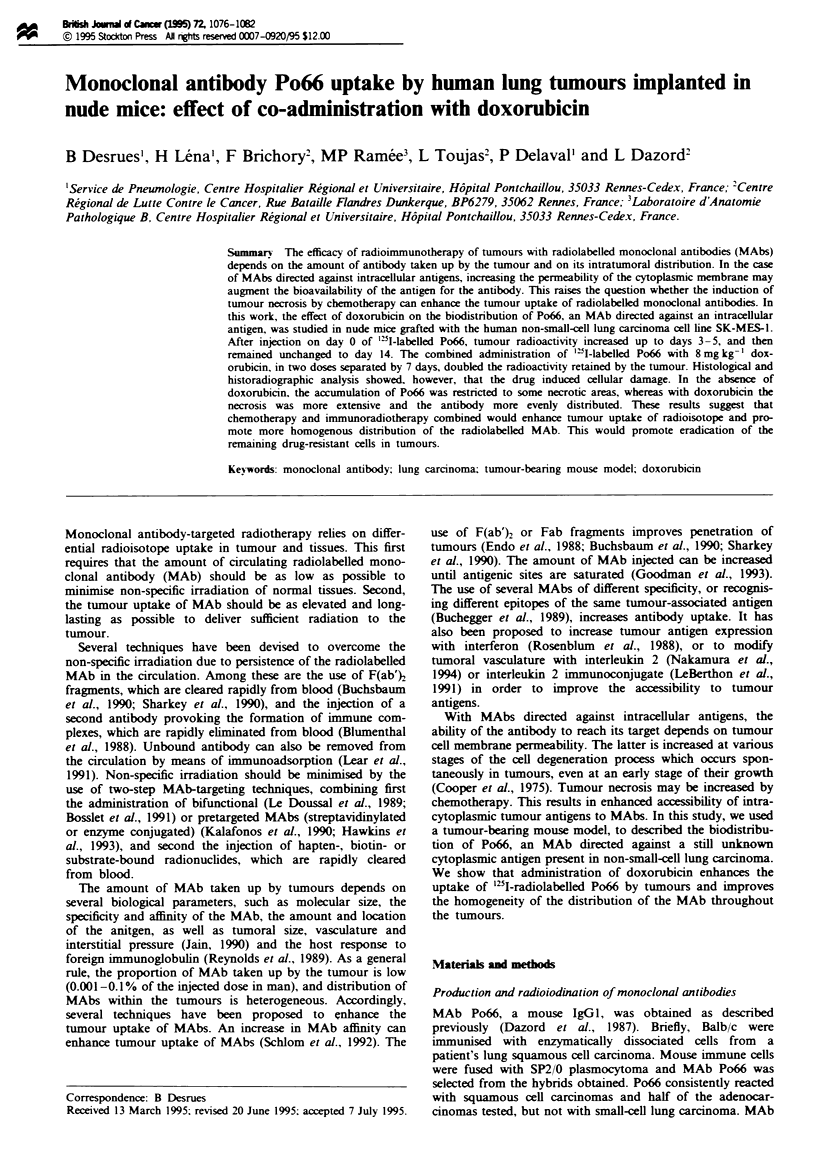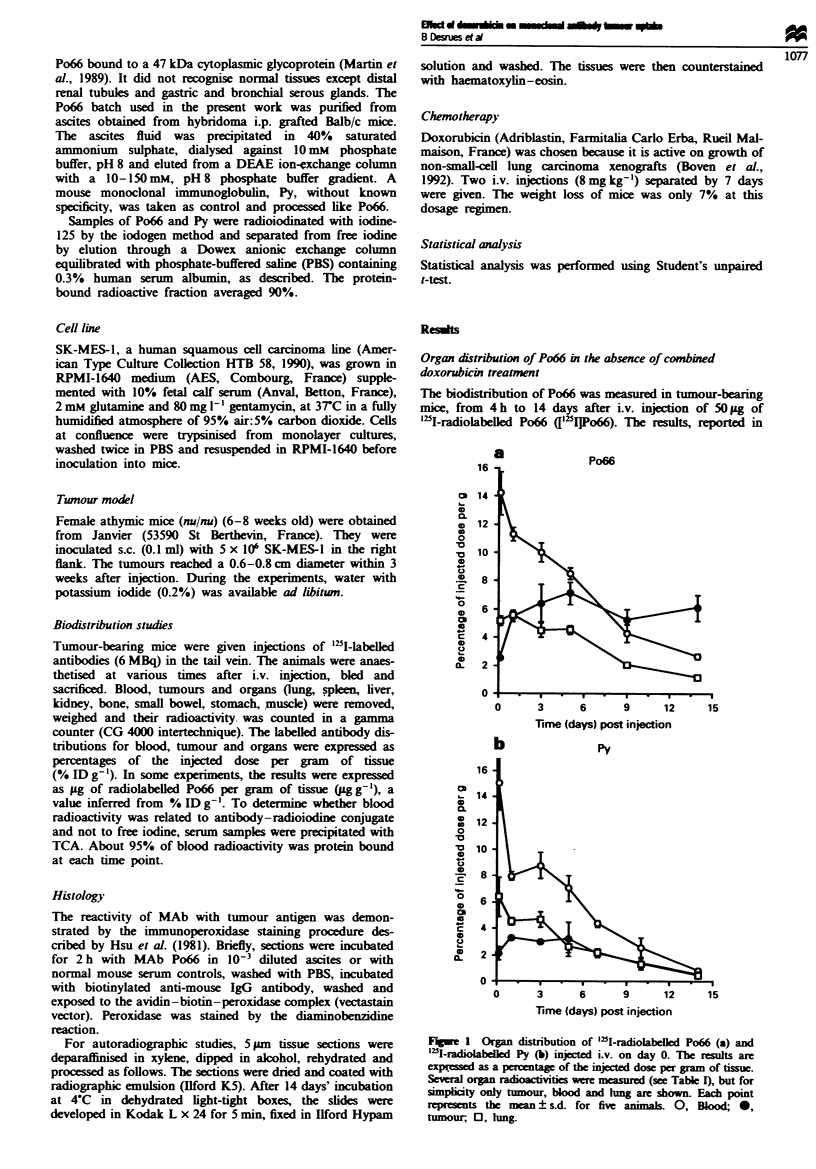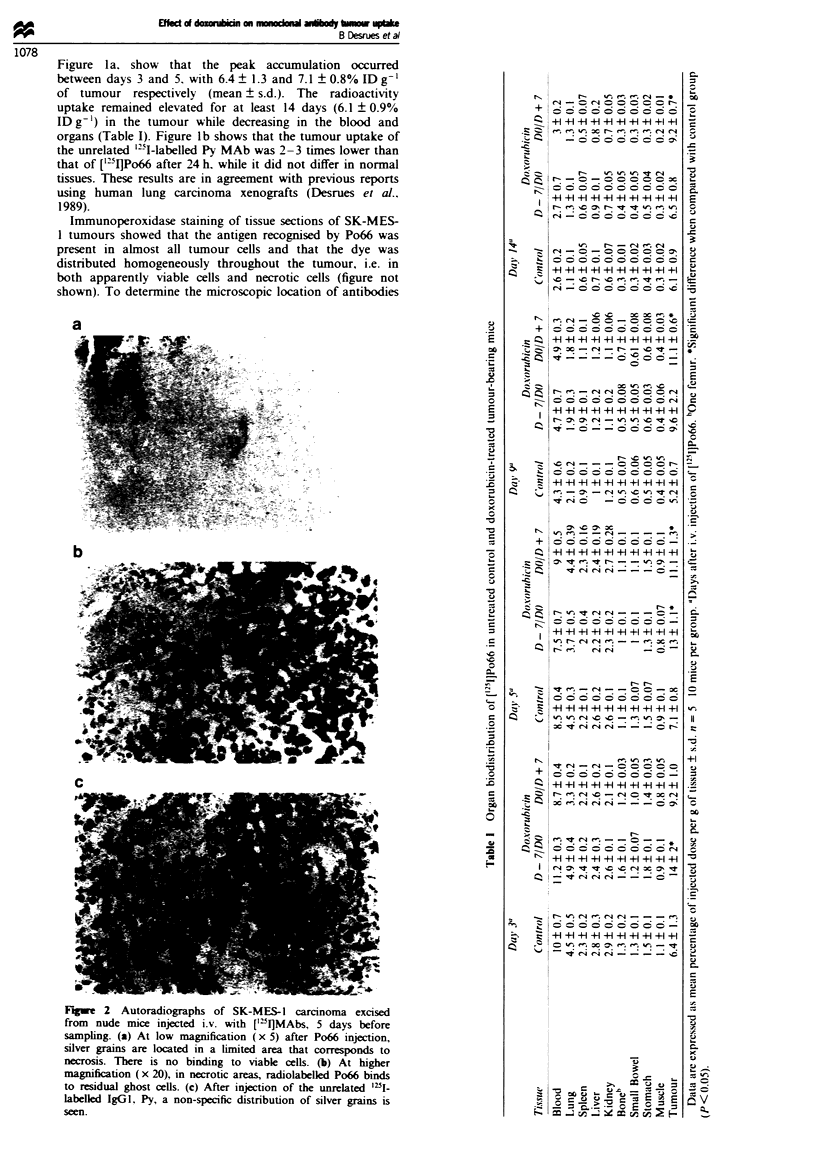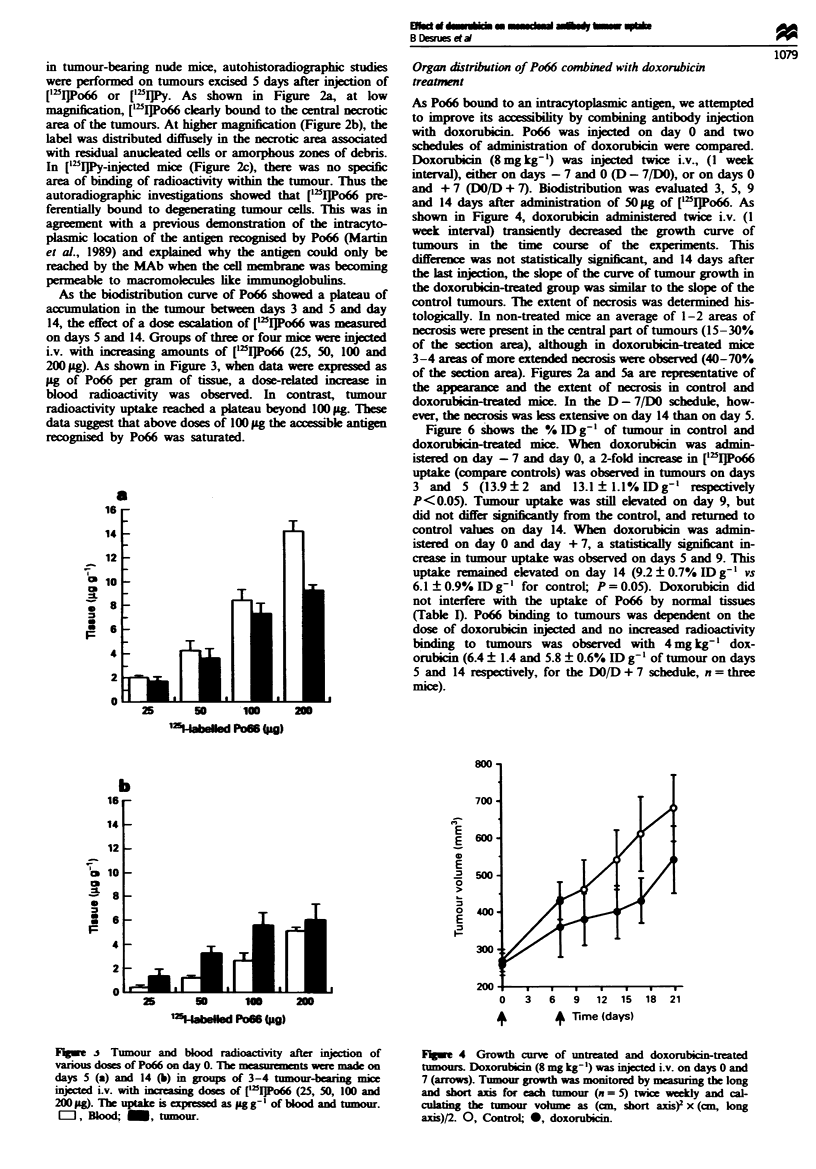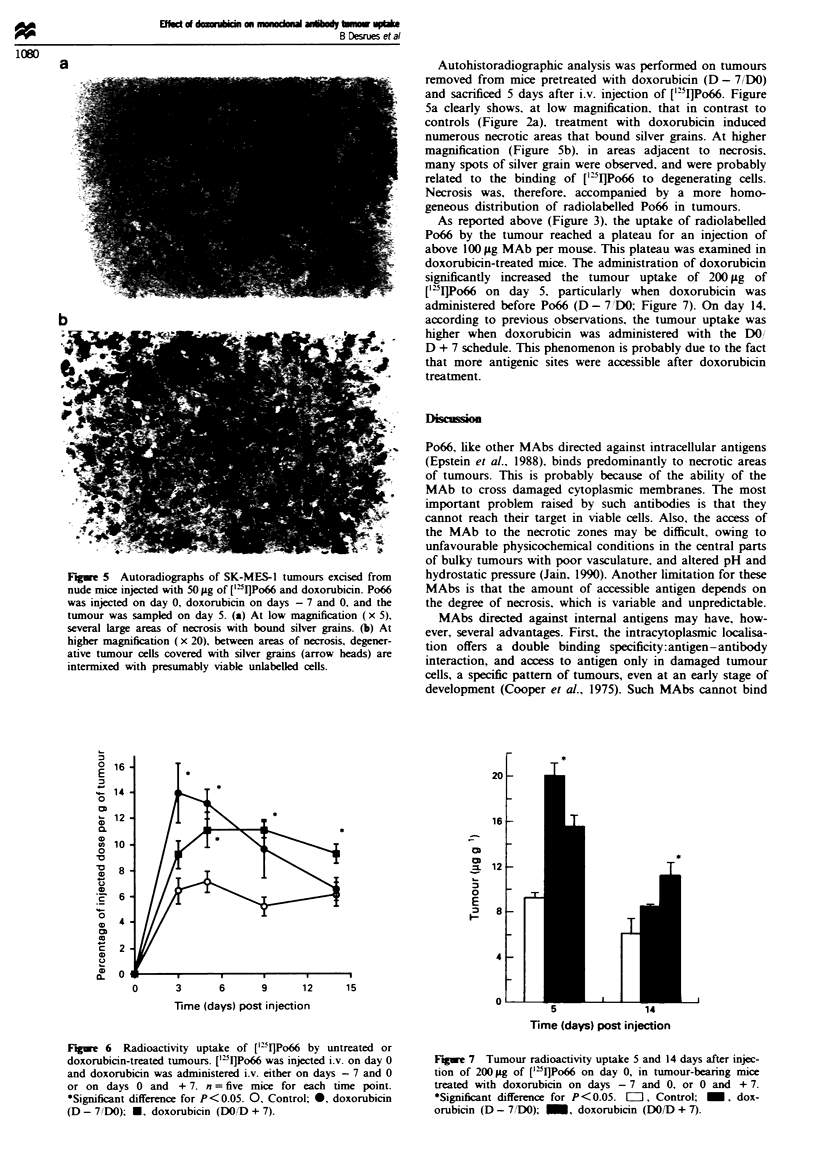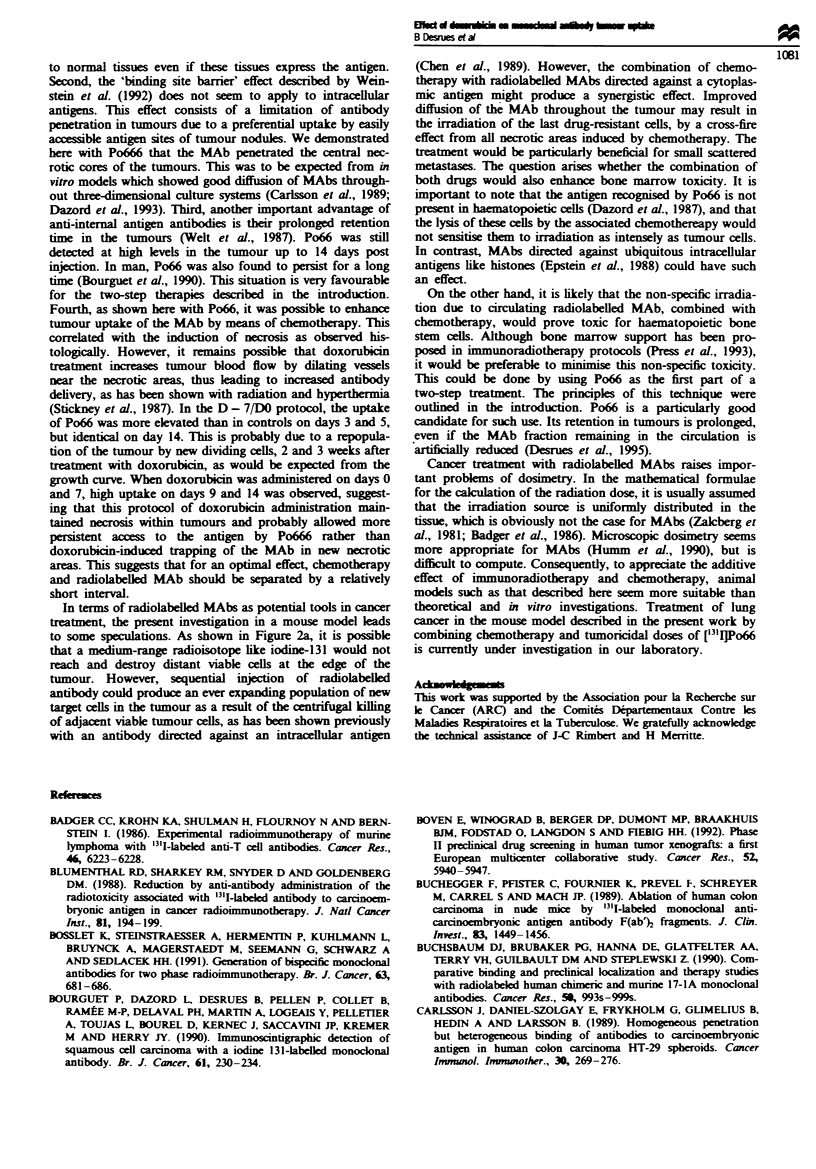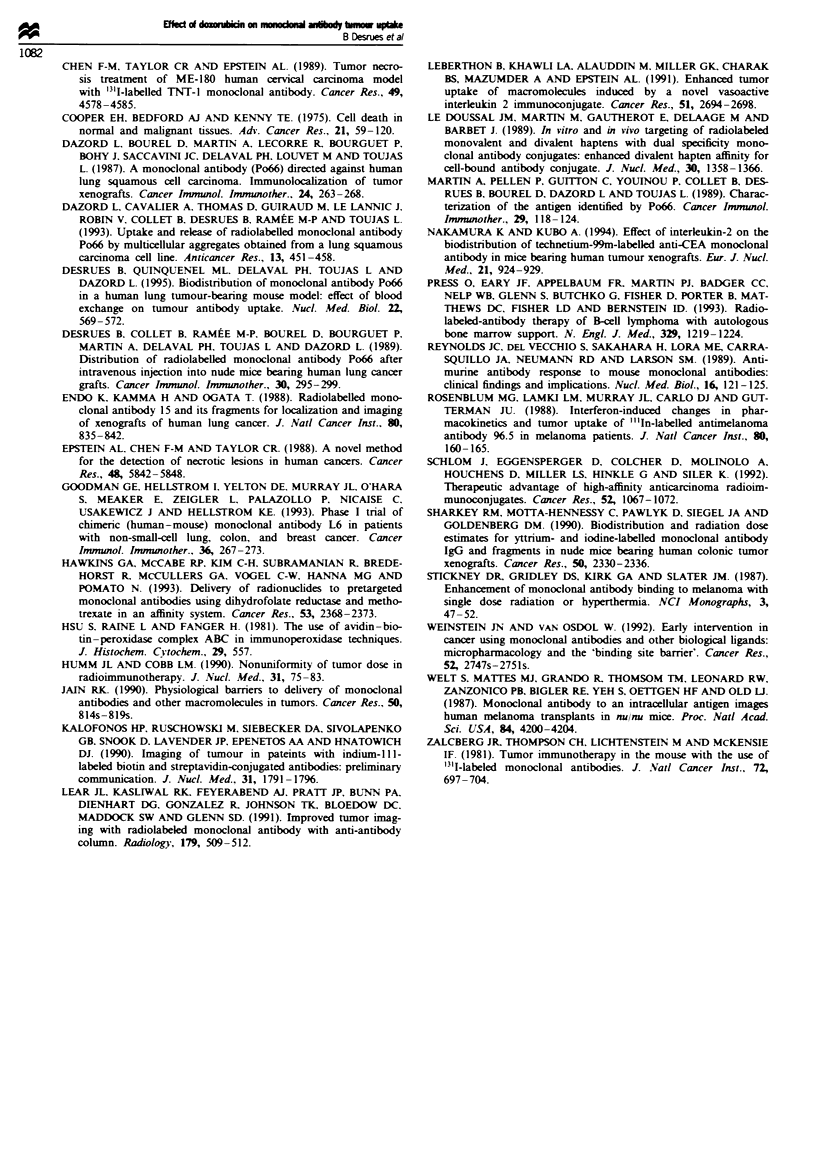# Monoclonal antibody Po66 uptake by human lung tumours implanted in nude mice: effect of co-administration with doxorubicin.

**DOI:** 10.1038/bjc.1995.468

**Published:** 1995-11

**Authors:** B. Desrues, H. Léna, F. Brichory, M. P. Ramée, L. Toujas, P. Delaval, L. Dazord

**Affiliations:** Service de Pneumologie, Centre Hospitalier Régional et Universitaire, Hôpital Pontchaillou, Rennes, France.

## Abstract

**Images:**


© 1995 Stockton Press All rights reserved 0007-0920/95 $12.00

### Monoclonal antibody Po66 uptake by human lung tumours implanted in nude mice: effect of co-administration with doxorubicin

B Desrues[1], H Léna[1], F Brichory[2], MP Ramée[3], L Toujas[2], P Delaval[1] and L Dazord[2]

[1]*Service de Pneumologie, Centre Hospitalier Régional et Universitaire, Hôpital Pontchaillou, 35033 Rennes-Cedex, France;* [2]*Centre Régional de Lutte Contre le Cancer, Rue Bataille Flandres Dunkerque, BP6279, 35062 Rennes, France;* [3]*Laboratoire d'Anatomie Pathologique B, Centre Hospitalier Régional et Universitaire, Hôpital Pontchaillou, 35033 Rennes-Cedex, France.*

**Summary** The efficacy of radioimmunotherapy of tumours with radiolabelled monoclonal antibodies (MAbs) depends on the amount of antibody taken up by the tumour and on its intratumoral distribution. In the case of MAbs directed against intracellular antigens, increasing the permeability of the cytoplasmic membrane may augment the bioavailability of the antigen for the antibody. This raises the question whether the induction of tumour necrosis by chemotherapy can enhance the tumour uptake of radiolabelled monoclonal antibodies. In this work, the effect of doxorubicin on the biodistribution of Po66, an MAb directed against an intracellular antigen, was studied in nude mice grafted with the human non-small-cell lung carcinoma cell line SK-MES-1. After injection on day 0 of $^{125}$I-labelled Po66, tumour radioactivity increased up to days 3-5, and then remained unchanged to day 14. The combined administration of $^{125}$I-labelled Po66 with 8 mg kg$^{-1}$ doxorubicin, in two doses separated by 7 days, doubled the radioactivity retained by the tumour. Histological and historadiographic analysis showed, however, that the drug induced cellular damage. In the absence of doxorubicin, the accumulation of Po66 was restricted to some necrotic areas, whereas with doxorubicin the necrosis was more extensive and the antibody more evenly distributed. These results suggest that chemotherapy and immunoradiotherapy combined would enhance tumour uptake of radioisotope and promote more homogenous distribution of the radiolabelled MAb. This would promote eradication of the remaining drug-resistant cells in tumours.

**Keywords**: monoclonal antibody; lung carcinoma; tumour-bearing mouse model; doxorubicin

Monoclonal antibody-targeted radiotherapy relies on differential radioisotope uptake in tumour and tissues. This first requires that the amount of circulating radiolabelled monoclonal antibody (MAb) should be as low as possible to minimise non-specific irradiation of normal tissues. Second, the tumour uptake of MAb should be as elevated and long-lasting as possible to deliver sufficient radiation to the tumour.

Several techniques have been devised to overcome the non-specific irradiation due to persistence of the radiolabelled MAb in the circulation. Among these are the use of F(ab')$_2$ fragments, which are cleared rapidly from blood (Buchsbaum *et al.*, 1990; Sharkey *et al.*, 1990), and the injection of a second antibody provoking the formation of immune complexes, which are rapidly eliminated from blood (Blumenthal *et al.*, 1988). Unbound antibody can also be removed from the circulation by means of immunoadsorption (Lear *et al.*, 1991). Non-specific irradiation should be minimised by the use of two-step MAb-targeting techniques, combining first the administration of bifunctional (Le Doussal *et al.*, 1989; Bosslet *et al.*, 1991) or pretargeted MAbs (streptavidinylated or enzyme conjugated) (Kalafonos *et al.*, 1990; Hawkins *et al.*, 1993), and second the injection of hapten-, biotin- or substrate-bound radionuclides, which are rapidly cleared from blood.

The amount of MAb taken up by tumours depends on several biological parameters, such as molecular size, the specificity and affinity of the MAb, the amount and location of the anitgen, as well as tumoral size, vasculature and interstitial pressure (Jain, 1990) and the host response to foreign immunoglobulin (Reynolds *et al.*, 1989). As a general rule, the proportion of MAb taken up by the tumour is low (0.001-0.1% of the injected dose in man), and distribution of MAbs within the tumours is heterogeneous. Accordingly, several techniques have been proposed to enhance the tumour uptake of MAbs. An increase in MAb affinity can enhance tumour uptake of MAbs (Schlom *et al.*, 1992). The use of F(ab')$_2$ or Fab fragments improves penetration of tumours (Endo *et al.*, 1988; Buchsbaum *et al.*, 1990; Sharkey *et al.*, 1990). The amount of MAb injected can be increased until antigenic sites are saturated (Goodman *et al.*, 1993). The use of several MAbs of different specificity, or recognising different epitopes of the same tumour-associated antigen (Buchegger *et al.*, 1989), increases antibody uptake. It has also been proposed to increase tumour antigen expression with interferon (Rosenblum *et al.*, 1988), or to modify tumoral vasculature with interleukin 2 (Nakamura *et al.*, 1994) or interleukin 2 immunoconjugate (LeBerthon *et al.*, 1991) in order to improve the accessibility to tumour antigens.

With MAbs directed against intracellular antigens, the ability of the antibody to reach its target depends on tumour cell membrane permeability. The latter is increased at various stages of the cell degeneration process which occurs spontaneously in tumours, even at an early stage of their growth (Cooper *et al.*, 1975). Tumour necrosis may be increased by chemotherapy. This results in enhanced accessibility of intracytoplasmic tumour antigens to MAbs. In this study, we used a tumour-bearing mouse model, to described the biodistribution of Po66, an MAb directed against a still unknown cytoplasmic antigen present in non-small-cell lung carcinoma. We show that administration of doxorubicin enhances the uptake of $^{125}$I-radiolabelled Po66 by tumours and improves the homogeneity of the distribution of the MAb throughout the tumours.

### Materials and methods

### *Production and radioiodination of monoclonal antibodies*

MAb Po66, a mouse IgG1, was obtained as described previously (Dazord *et al.*, 1987). Briefly, Balb/c were immunised with enzymatically dissociated cells from a patient's lung squamous cell carcinoma. Mouse immune cells were fused with SP2/0 plasmocytoma and MAb Po66 was selected from the hybrids obtained. Po66 consistently reacted with squamous cell carcinomas and half of the adenocarcinomas tested, but not with small-cell lung carcinoma. MAb

Correspondence: B Desrues
Received 13 March 1995; revised 20 June 1995; accepted 7 July 1995.

Po66 bound to a 47 kDa cytoplasmic glycoprotein (Martin *et al.*, 1989). It did not recognise normal tissues except distal renal tubules and gastric and bronchial serous glands. The Po66 batch used in the present work was purified from ascites obtained from hybridoma i.p. grafted Balb/c mice. The ascites fluid was precipitated in 40% saturated ammonium sulphate, dialysed against 10 mM phosphate buffer, pH 8 and eluted from a DEAE ion-exchange column with a 10–150 mM, pH 8 phosphate buffer gradient. A mouse monoclonal immunoglobulin, Py, without known specificity, was taken as control and processed like Po66.

Samples of Po66 and Py were radioiodinated with iodine-125 by the iodogen method and separated from free iodine by elution through a Dowex anionic exchange column equilibrated with phosphate-buffered saline (PBS) containing 0.3% human serum albumin, as described. The protein-bound radioactive fraction averaged 90%.

### *Cell line*

SK-MES-1, a human squamous cell carcinoma line (American Type Culture Collection HTB 58, 1990), was grown in RPMI-1640 medium (AES, Combourg, France) supplemented with 10% fetal calf serum (Anval, Betton, France), 2 mM glutamine and 80 mg $l^{-1}$ gentamycin, at 37°C in a fully humidified atmosphere of 95% air:5% carbon dioxide. Cells at confluence were trypsinised from monolayer cultures, washed twice in PBS and resuspended in RPMI-1640 before inoculation into mice.

### *Tumour model*

Female athymic mice (*nu/nu*) (6–8 weeks old) were obtained from Janvier (53590 St Berthevin, France). They were inoculated s.c. (0.1 ml) with $5 \times 10^6$ SK-MES-1 in the right flank. The tumours reached a 0.6–0.8 cm diameter within 3 weeks after injection. During the experiments, water with potassium iodide (0.2%) was available *ad libitum*.

### *Biodistribution studies*

Tumour-bearing mice were given injections of $^{125}$I-labelled antibodies (6 MBq) in the tail vein. The animals were anaesthetised at various times after i.v. injection, bled and sacrificed. Blood, tumours and organs (lung, spleen, liver, kidney, bone, small bowel, stomach, muscle) were removed, weighed and their radioactivity was counted in a gamma counter (CG 4000 intertechnique). The labelled antibody distributions for blood, tumour and organs were expressed as percentages of the injected dose per gram of tissue (% ID $g^{-1}$). In some experiments, the results were expressed as $\mu$g of radiolabelled Po66 per gram of tissue ($\mu g\ g^{-1}$), a value inferred from % ID $g^{-1}$. To determine whether blood radioactivity was related to antibody–radioiodine conjugate and not to free iodine, serum samples were precipitated with TCA. About 95% of blood radioactivity was protein bound at each time point.

### *Histology*

The reactivity of MAb with tumour antigen was demonstrated by the immunoperoxidase staining procedure described by Hsu *et al.* (1981). Briefly, sections were incubated for 2 h with MAb Po66 in $10^{-3}$ diluted ascites or with normal mouse serum controls, washed with PBS, incubated with biotinylated anti-mouse IgG antibody, washed and exposed to the avidin–biotin–peroxidase complex (vectastain vector). Peroxidase was stained by the diaminobenzidine reaction.

For autoradiographic studies, 5 $\mu$m tissue sections were deparaffinised in xylene, dipped in alcohol, rehydrated and processed as follows. The sections were dried and coated with radiographic emulsion (Ilford K5). After 14 days' incubation at 4°C in dehydrated light-tight boxes, the slides were developed in Kodak L × 24 for 5 min, fixed in Ilford Hypam solution and washed. The tissues were then counterstained with haematoxylin–eosin.

### *Chemotherapy*

Doxorubicin (Adriblastin, Farmitalia Carlo Erba, Rueil Malmaison, France) was chosen because it is active on growth of non-small-cell lung carcinoma xenografts (Boven *et al.*, 1992). Two i.v. injections (8 mg $kg^{-1}$) separated by 7 days were given. The weight loss of mice was only 7% at this dosage regimen.

### *Statistical analysis*

Statistical analysis was performed using Student's unpaired *t*-test.

### Results

### *Organ distribution of Po66 in the absence of combined doxorubicin treatment*

The biodistribution of Po66 was measured in tumour-bearing mice, from 4 h to 14 days after i.v. injection of 50 $\mu$g of $^{125}$I-radiolabelled Po66 ([$^{125}$I]Po66). The results, reported in

**Figure 1** Organ distribution of $^{125}$I-radiolabelled Po66 (**a**) and $^{125}$I-radiolabelled Py (**b**) injected i.v. on day 0. The results are expressed as a percentage of the injected dose per gram of tissue. Several organ radioactivities were measured (see Table I), but for simplicity only tumour, blood and lung are shown. Each point represents the mean ± s.d. for five animals. ○, Blood; ●, tumour; □, lung.

Figure 1a, show that the peak accumulation occurred between days 3 and 5, with 6.4 ± 1.3 and 7.1 ± 0.8% ID $g^{-1}$ of tumour respectively (mean ± s.d.). The radioactivity uptake remained elevated for at least 14 days (6.1 ± 0.9% ID $g^{-1}$) in the tumour while decreasing in the blood and organs (Table I). Figure 1b shows that the tumour uptake of the unrelated $^{125}$I-labelled Py MAb was 2–3 times lower than that of [$^{125}$I]Po66 after 24 h, while it did not differ in normal tissues. These results are in agreement with previous reports using human lung carcinoma xenografts (Desrues *et al*., 1989).

Immunoperoxidase staining of tissue sections of SK-MES-1 tumours showed that the antigen recognised by Po66 was present in almost all tumour cells and that the dye was distributed homogeneously throughout the tumour, i.e. in both apparently viable cells and necrotic cells (figure not shown). To determine the microscopic location of antibodies

**Figure 2** Autoradiographs of SK-MES-1 carcinoma excised from nude mice injected i.v. with [$^{125}$I]MAbs, 5 days before sampling. (**a**) At low magnification (× 5) after Po66 injection, silver grains are located in a limited area that corresponds to necrosis. There is no binding to viable cells. (**b**) At higher magnification (× 20), in necrotic areas, radiolabelled Po66 binds to residual ghost cells. (**c**) After injection of the unrelated $^{125}$I-labelled IgG1, Py, a non-specific distribution of silver grains is seen.

**Table I** Organ biodistribution of [$^{125}$I]Po66 in untreated control and doxorubicin-treated tumour-bearing mice

| | *Day 3*[a] | | | *Day 5*[a] | | | *Day 9*[a] | | | *Day 14*[a] | | |
|---|---|---|---|---|---|---|---|---|---|---|---|---|
| | | *Doxorubicin* | | | *Doxorubicin* | | | *Doxorubicin* | | | *Doxorubicin* | |
| *Tissue* | *Control* | *D – 7/D0* | *D0/D + 7* | *Control* | *D – 7/D0* | *D0/D + 7* | *Control* | *D – 7/D0* | *D0/D + 7* | *Control* | *D – 7/D0* | *D0/D + 7* |
| Blood | 10 ± 0.7 | 11.2 ± 0.3 | 8.7 ± 0.4 | 8.5 ± 0.4 | 7.5 ± 0.7 | 9 ± 0.5 | 4.3 ± 0.6 | 4.7 ± 0.7 | 4.9 ± 0.3 | 2.6 ± 0.2 | 2.7 ± 0.7 | 3 ± 0.2 |
| Lung | 4.5 ± 0.5 | 4.9 ± 0.4 | 3.3 ± 0.2 | 4.5 ± 0.3 | 3.7 ± 0.5 | 4.4 ± 0.39 | 2.1 ± 0.2 | 1.9 ± 0.3 | 1.8 ± 0.2 | 1.1 ± 0.1 | 1.3 ± 0.1 | 1.3 ± 0.1 |
| Spleen | 2.3 ± 0.2 | 2.4 ± 0.2 | 2.2 ± 0.1 | 2.2 ± 0.1 | 2 ± 0.4 | 2.3 ± 0.16 | 0.9 ± 0.1 | 0.9 ± 0.1 | 1.1 ± 0.1 | 0.6 ± 0.05 | 0.6 ± 0.07 | 0.5 ± 0.07 |
| Liver | 2.8 ± 0.3 | 2.4 ± 0.3 | 2.6 ± 0.2 | 2.6 ± 0.2 | 2.2 ± 0.2 | 2.4 ± 0.19 | 1 ± 0.1 | 1.2 ± 0.2 | 1.2 ± 0.06 | 0.7 ± 0.1 | 0.9 ± 0.1 | 0.8 ± 0.2 |
| Kidney | 2.9 ± 0.2 | 2.6 ± 0.1 | 2.1 ± 0.1 | 2.6 ± 0.1 | 2.3 ± 0.2 | 2.7 ± 0.28 | 1.2 ± 0.1 | 1.1 ± 0.2 | 1.1 ± 0.06 | 0.6 ± 0.07 | 0.7 ± 0.05 | 0.7 ± 0.05 |
| Bone[b] | 1.3 ± 0.2 | 1.6 ± 0.1 | 1.2 ± 0.03 | 1.1 ± 0.1 | 1 ± 0.1 | 1.1 ± 0.1 | 0.5 ± 0.07 | 0.5 ± 0.08 | 0.7 ± 0.1 | 0.3 ± 0.01 | 0.4 ± 0.05 | 0.3 ± 0.03 |
| Small Bowel | 1.3 ± 0.1 | 1.2 ± 0.07 | 1.0 ± 0.05 | 1.3 ± 0.07 | 1 ± 0.1 | 1.1 ± 0.1 | 0.6 ± 0.06 | 0.5 ± 0.05 | 0.61 ± 0.08 | 0.3 ± 0.02 | 0.4 ± 0.05 | 0.3 ± 0.03 |
| Stomach | 1.5 ± 0.1 | 1.8 ± 0.1 | 1.4 ± 0.03 | 1.5 ± 0.07 | 1.3 ± 0.1 | 1.5 ± 0.1 | 0.5 ± 0.05 | 0.6 ± 0.03 | 0.6 ± 0.08 | 0.4 ± 0.03 | 0.5 ± 0.04 | 0.3 ± 0.02 |
| Muscle | 1.1 ± 0.1 | 0.9 ± 0.1 | 0.8 ± 0.05 | 0.9 ± 0.1 | 0.8 ± 0.07 | 0.9 ± 0.1 | 0.4 ± 0.05 | 0.4 ± 0.06 | 0.4 ± 0.03 | 0.3 ± 0.02 | 0.3 ± 0.02 | 0.2 ± 0.01 |
| Tumour | 6.4 ± 1.3 | 14 ± 2* | 9.2 ± 1.0 | 7.1 ± 0.8 | 13 ± 1.1* | 11.1 ± 1.3* | 5.2 ± 0.7 | 9.6 ± 2.2 | 11.1 ± 0.6* | 6.1 ± 0.9 | 6.5 ± 0.8 | 9.2 ± 0.7* |

Data are expressed as mean percentage of injected dose per g of tissue ± s.d. $n = 5$ 10 mice per group. [a]Days after i.v. injection of [$^{125}$I]Po66. [b]One femur. *Significant difference when compared with control group ($P < 0.05$).

in tumour-bearing nude mice, autohistoradiographic studies were performed on tumours excised 5 days after injection of [$^{125}$I]Po66 or [$^{125}$I]Py. As shown in Figure 2a, at low magnification, [$^{125}$I]Po66 clearly bound to the central necrotic area of the tumours. At higher magnification (Figure 2b), the label was distributed diffusely in the necrotic area associated with residual anucleated cells or amorphous zones of debris. In [$^{125}$I]Py-injected mice (Figure 2c), there was no specific area of binding of radioactivity within the tumour. Thus the autoradiographic investigations showed that [$^{125}$I]Po66 preferentially bound to degenerating tumour cells. This was in agreement with a previous demonstration of the intracytoplasmic location of the antigen recognised by Po66 (Martin *et al.*, 1989) and explained why the antigen could only be reached by the MAb when the cell membrane was becoming permeable to macromolecules like immunoglobulins.

As the biodistribution curve of Po66 showed a plateau of accumulation in the tumour between days 3 and 5 and day 14, the effect of a dose escalation of [$^{125}$I]Po66 was measured on days 5 and 14. Groups of three or four mice were injected i.v. with increasing amounts of [$^{125}$I]Po66 (25, 50, 100 and 200 μg). As shown in Figure 3, when data were expressed as μg of Po66 per gram of tissue, a dose-related increase in blood radioactivity was observed. In contrast, tumour radioactivity uptake reached a plateau beyond 100 μg. These data suggest that above doses of 100 μg the accessible antigen recognised by Po66 was saturated.

**Figure 3** Tumour and blood radioactivity after injection of various doses of Po66 on day 0. The measurements were made on days 5 (**a**) and 14 (**b**) in groups of 3–4 tumour-bearing mice injected i.v. with increasing doses of [$^{125}$I]Po66 (25, 50, 100 and 200 μg). The uptake is expressed as μg $g^{-1}$ of blood and tumour. ▭, Blood; ■, tumour.

*Organ distribution of Po66 combined with doxorubicin treatment*

As Po66 bound to an intracytoplasmic antigen, we attempted to improve its accessibility by combining antibody injection with doxorubicin. Po66 was injected on day 0 and two schedules of administration of doxorubicin were compared. Doxorubicin (8 mg $kg^{-1}$) was injected twice i.v., (1 week interval), either on days − 7 and 0 (D − 7/D0), or on days 0 and + 7 (D0/D + 7). Biodistribution was evaluated 3, 5, 9 and 14 days after administration of 50 μg of [$^{125}$I]Po66. As shown in Figure 4, doxorubicin administered twice i.v. (1 week interval) transiently decreased the growth curve of tumours in the time course of the experiments. This difference was not statistically significant, and 14 days after the last injection, the slope of the curve of tumour growth in the doxorubicin-treated group was similar to the slope of the control tumours. The extent of necrosis was determined histologically. In non-treated mice an average of 1–2 areas of necrosis were present in the central part of tumours (15–30% of the section area), although in doxorubicin-treated mice 3–4 areas of more extended necrosis were observed (40–70% of the section area). Figures 2a and 5a are representative of the appearance and the extent of necrosis in control and doxorubicin-treated mice. In the D − 7/D0 schedule, however, the necrosis was less extensive on day 14 than on day 5.

Figure 6 shows the % ID $g^{-1}$ of tumour in control and doxorubicin-treated mice. When doxorubicin was administered on day − 7 and day 0, a 2-fold increase in [$^{125}$I]Po66 uptake (compare controls) was observed in tumours on days 3 and 5 (13.9 ± 2 and 13.1 ± 1.1% ID $g^{-1}$ respectively $P < 0.05$). Tumour uptake was still elevated on day 9, but did not differ significantly from the control, and returned to control values on day 14. When doxorubicin was administered on day 0 and day + 7, a statistically significant increase in tumour uptake was observed on days 5 and 9. This uptake remained elevated on day 14 (9.2 ± 0.7% ID $g^{-1}$ *vs* 6.1 ± 0.9% ID $g^{-1}$ for control; $P = 0.05$). Doxorubicin did not interfere with the uptake of Po66 by normal tissues (Table I). Po66 binding to tumours was dependent on the dose of doxorubicin injected and no increased radioactivity binding to tumours was observed with 4 mg $kg^{-1}$ doxorubicin (6.4 ± 1.4 and 5.8 ± 0.6% ID $g^{-1}$ of tumour on days 5 and 14 respectively, for the D0/D + 7 schedule, $n$ = three mice).

**Figure 4** Growth curve of untreated and doxorubicin-treated tumours. Doxorubicin (8 mg $kg^{-1}$) was injected i.v. on days 0 and 7 (arrows). Tumour growth was monitored by measuring the long and short axis for each tumour ($n$ = 5) twice weekly and calculating the tumour volume as (cm, short axis)$^2$ × (cm, long axis)/2. ○, Control; ●, doxorubicin.

**Figure 5** Autoradiographs of SK-MES-1 tumours excised from nude mice injected with 50 µg of [[125]I]Po66 and doxorubicin. Po66 was injected on day 0, doxorubicin on days − 7 and 0, and the tumour was sampled on day 5. (**a**) At low magnification ( × 5), several large areas of necrosis with bound silver grains. (**b**) At higher magnification ( × 20), between areas of necrosis, degenerative tumour cells covered with silver grains (arrow heads) are intermixed with presumably viable unlabelled cells.

Autohistoradiographic analysis was performed on tumours removed from mice pretreated with doxorubicin (D − 7/D0) and sacrificed 5 days after i.v. injection of [[125]I]Po66. Figure 5a clearly shows, at low magnification, that in contrast to controls (Figure 2a), treatment with doxorubicin induced numerous necrotic areas that bound silver grains. At higher magnification (Figure 5b), in areas adjacent to necrosis, many spots of silver grain were observed, and were probably related to the binding of [[125]I]Po66 to degenerating cells. Necrosis was, therefore, accompanied by a more homogeneous distribution of radiolabelled Po66 in tumours.

As reported above (Figure 3), the uptake of radiolabelled Po66 by the tumour reached a plateau for an injection of above 100 µg MAb per mouse. This plateau was examined in doxorubicin-treated mice. The administration of doxorubicin significantly increased the tumour uptake of 200 µg of [[125]I]Po66 on day 5, particularly when doxorubicin was administered before Po66 (D − 7/D0; Figure 7). On day 14, according to previous observations, the tumour uptake was higher when doxorubicin was administered with the D0/D + 7 schedule. This phenomenon is probably due to the fact that more antigenic sites were accessible after doxorubicin treatment.

### Discussion

Po66, like other MAbs directed against intracellular antigens (Epstein *et al.*, 1988), binds predominantly to necrotic areas of tumours. This is probably because of the ability of the MAb to cross damaged cytoplasmic membranes. The most important problem raised by such antibodies is that they cannot reach their target in viable cells. Also, the access of the MAb to the necrotic zones may be difficult, owing to unfavourable physicochemical conditions in the central parts of bulky tumours with poor vasculature, and altered pH and hydrostatic pressure (Jain, 1990). Another limitation for these MAbs is that the amount of accessible antigen depends on the degree of necrosis, which is variable and unpredictable.

MAbs directed against internal antigens may have, however, several advantages. First, the intracytoplasmic localisation offers a double binding specificity: antigen–antibody interaction, and access to antigen only in damaged tumour cells, a specific pattern of tumours, even at an early stage of development (Cooper *et al.*, 1975). Such MAbs cannot bind

**Figure 6** Radioactivity uptake of [[125]I]Po66 by untreated or doxorubicin-treated tumours. [[125]I]Po66 was injected i.v. on day 0 and doxorubicin was administered i.v. either on days − 7 and 0 or on days 0 and + 7. *n* = five mice for each time point. *Significant difference for $P < 0.05$. O, Control; ●, doxorubicin (D − 7/D0); ■, doxorubicin (D0/D + 7).

**Figure 7** Tumour radioactivity uptake 5 and 14 days after injection of 200 µg of [[125]I]Po66 on day 0, in tumour-bearing mice treated with doxorubicin on days − 7 and 0, or 0 and + 7. *Significant difference for $P < 0.05$. □, Control; ■, doxorubicin (D − 7/D0); ▩, doxorubicin (D0/D + 7).

to normal tissues even if these tissues express the antigen. Second, the 'binding site barrier' effect described by Weinstein *et al.* (1992) does not seem to apply to intracellular antigens. This effect consists of a limitation of antibody penetration in tumours due to a preferential uptake by easily accessible antigen sites of tumour nodules. We demonstrated here with Po666 that the MAb penetrated the central necrotic cores of the tumours. This was to be expected from *in vitro* models which showed good diffusion of MAbs throughout three-dimensional culture systems (Carlsson *et al.*, 1989; Dazord *et al.*, 1993). Third, another important advantage of anti-internal antigen antibodies is their prolonged retention time in the tumours (Welt *et al.*, 1987). Po66 was still detected at high levels in the tumour up to 14 days post injection. In man, Po66 was also found to persist for a long time (Bourguet *et al.*, 1990). This situation is very favourable for the two-step therapies described in the introduction. Fourth, as shown here with Po66, it was possible to enhance tumour uptake of the MAb by means of chemotherapy. This correlated with the induction of necrosis as observed histologically. However, it remains possible that doxorubicin treatment increases tumour blood flow by dilating vessels near the necrotic areas, thus leading to increased antibody delivery, as has been shown with radiation and hyperthermia (Stickney *et al.*, 1987). In the D − 7/D0 protocol, the uptake of Po66 was more elevated than in controls on days 3 and 5, but identical on day 14. This is probably due to a repopulation of the tumour by new dividing cells, 2 and 3 weeks after treatment with doxorubicin, as would be expected from the growth curve. When doxorubicin was administered on days 0 and 7, high uptake on days 9 and 14 was observed, suggesting that this protocol of doxorubicin administration maintained necrosis within tumours and probably allowed more persistent access to the antigen by Po666 rather than doxorubicin-induced trapping of the MAb in new necrotic areas. This suggests that for an optimal effect, chemotherapy and radiolabelled MAb should be separated by a relatively short interval.

In terms of radiolabelled MAbs as potential tools in cancer treatment, the present investigation in a mouse model leads to some speculations. As shown in Figure 2a, it is possible that a medium-range radioisotope like iodine-131 would not reach and destroy distant viable cells at the edge of the tumour. However, sequential injection of radiolabelled antibody could produce an ever expanding population of new target cells in the tumour as a result of the centrifugal killing of adjacent viable tumour cells, as has been shown previously with an antibody directed against an intracellular antigen (Chen *et al.*, 1989). However, the combination of chemotherapy with radiolabelled MAbs directed against a cytoplasmic antigen might produce a synergistic effect. Improved diffusion of the MAb throughout the tumour may result in the irradiation of the last drug-resistant cells, by a cross-fire effect from all necrotic areas induced by chemotherapy. The treatment would be particularly beneficial for small scattered metastases. The question arises whether the combination of both drugs would also enhance bone marrow toxicity. It is important to note that the antigen recognised by Po66 is not present in haematopoietic cells (Dazord *et al.*, 1987), and that the lysis of these cells by the associated chemothereapy would not sensitise them to irradiation as intensely as tumour cells. In contrast, MAbs directed against ubiquitous intracellular antigens like histones (Epstein *et al.*, 1988) could have such an effect.

On the other hand, it is likely that the non-specific irradiation due to circulating radiolabelled MAb, combined with chemotherapy, would prove toxic for haematopoietic bone stem cells. Although bone marrow support has been proposed in immunoradiotherapy protocols (Press *et al.*, 1993), it would be preferable to minimise this non-specific toxicity. This could be done by using Po66 as the first part of a two-step treatment. The principles of this technique were outlined in the introduction. Po66 is a particularly good candidate for such use. Its retention in tumours is prolonged, even if the MAb fraction remaining in the circulation is artificially reduced (Desrues *et al.*, 1995).

Cancer treatment with radiolabelled MAbs raises important problems of dosimetry. In the mathematical formulae for the calculation of the radiation dose, it is usually assumed that the irradiation source is uniformly distributed in the tissue, which is obviously not the case for MAbs (Zalcberg *et al.*, 1981; Badger *et al.*, 1986). Microscopic dosimetry seems more appropriate for MAbs (Humm *et al.*, 1990), but is difficult to compute. Consequently, to appreciate the additive effect of immunoradiotherapy and chemotherapy, animal models such as that described here seem more suitable than theoretical and *in vitro* investigations. Treatment of lung cancer in the mouse model described in the present work by combining chemotherapy and tumoricidal doses of [$^{131}$I]Po66 is currently under investigation in our laboratory.

**Acknowledgements**

This work was supported by the Association pour la Recherche sur le Cancer (ARC) and the Comités Départementaux Contre les Maladies Respiratoires et la Tuberculose. We gratefully acknowledge the technical assistance of J-C Rimbert and H Merritte.

**References**

BADGER CC, KROHN KA, SHULMAN H, FLOURNOY N AND BERNSTEIN I. (1986). Experimental radioimmunotherapy of murine lymphoma with $^{131}$I-labeled anti-T cell antibodies. *Cancer Res.*, **46**, 6223-6228.

BLUMENTHAL RD, SHARKEY RM, SNYDER D AND GOLDENBERG DM. (1988). Reduction by anti-antibody administration of the radiotoxicity associated with $^{131}$I-labeled antibody to carcinoembryonic antigen in cancer radioimmunotherapy. *J. Natl Cancer Inst.*, **81**, 194-199.

BOSSLET K, STEINSTRAESSER A, HERMENTIN P, KUHLMANN L, BRUYNCK A, MAGERSTAEDT M, SEEMANN G, SCHWARZ A AND SEDLACEK HH. (1991). Generation of bispecific monoclonal antibodies for two phase radioimmunotherapy. *Br. J. Cancer*, **63**, 681-686.

BOURGUET P, DAZORD L, DESRUES B, PELLEN P, COLLET B, RAMÉE M-P, DELAVAL PH, MARTIN A, LOGEAIS Y, PELLETIER A, TOUJAS L, BOUREL D, KERNEC J, SACCAVINI JP, KREMER M AND HERRY JY. (1990). Immunoscintigraphic detection of squamous cell carcinoma with a iodine 131-labelled monoclonal antibody. *Br. J. Cancer*, **61**, 230-234.

BOVEN E, WINOGRAD B, BERGER DP, DUMONT MP, BRAAKHUIS BJM, FODSTAD O, LANGDON S AND FIEBIG HH. (1992). Phase II preclinical drug screening in human tumor xenografts: a first European multicenter collaborative study. *Cancer Res.*, **52**, 5940-5947.

BUCHEGGER F, PFISTER C, FOURNIER K, PREVEL F, SCHREYER M, CARREL S AND MACH JP. (1989). Ablation of human colon carcinoma in nude mice by $^{131}$I-labeled monoclonal anti-carcinoembryonic antigen antibody F(ab')$_2$ fragments. *J. Clin. Invest.*, **83**, 1449-1456.

BUCHSBAUM DJ, BRUBAKER PG, HANNA DE, GLATFELTER AA, TERRY VH, GUILBAULT DM AND STEPLEWSKI Z. (1990). Comparative binding and preclinical localization and therapy studies with radiolabeled human chimeric and murine 17-1A monoclonal antibodies. *Cancer Res.*, **50**, 993s-999s.

CARLSSON J, DANIEL-SZOLGAY E, FRYKHOLM G, GLIMELIUS B, HEDIN A AND LARSSON B. (1989). Homogeneous penetration but heterogeneous binding of antibodies to carcinoembryonic antigen in human colon carcinoma HT-29 spheroids. *Cancer Immunol. Immunother.*, **30**, 269-276.

CHEN F-M, TAYLOR CR AND EPSTEIN AL. (1989). Tumor necrosis treatment of ME-180 human cervical carcinoma model with $^{131}$I-labelled TNT-1 monoclonal antibody. *Cancer Res.*, **49**, 4578-4585.

COOPER EH, BEDFORD AJ AND KENNY TE. (1975). Cell death in normal and malignant tissues. *Adv. Cancer Res.*, **21**, 59-120.

DAZORD L, BOUREL D, MARTIN A, LECORRE R, BOURGUET P, BOHY J, SACCAVINI JC, DELAVAL PH, LOUVET M AND TOUJAS L. (1987). A monoclonal antibody (Po66) directed against human lung squamous cell carcinoma. Immunolocalization of tumor xenografts. *Cancer Immunol. Immunother.*, **24**, 263-268.

DAZORD L, CAVALIER A, THOMAS D, GUIRAUD M, LE LANNIC J, ROBIN V, COLLET B, DESRUES B, RAMÉE M-P AND TOUJAS L. (1993). Uptake and release of radiolabelled monoclonal antibody Po66 by multicellular aggregates obtained from a lung squamous carcinoma cell line. *Anticancer Res.*, **13**, 451-458.

DESRUES B, QUINQUENEL ML, DELAVAL PH, TOUJAS L AND DAZORD L. (1995). Biodistribution of monoclonal antibody Po66 in a human lung tumour-bearing mouse model: effect of blood exchange on tumour antibody uptake. *Nucl. Med. Biol.* **22**, 569-572.

DESRUES B, COLLET B, RAMÉE M-P, BOUREL D, BOURGUET P, MARTIN A, DELAVAL PH, TOUJAS L AND DAZORD L. (1989). Distribution of radiolabelled monoclonal antibody Po66 after intravenous injection into nude mice bearing human lung cancer grafts. *Cancer Immunol. Immunother.*, **30**, 295-299.

ENDO K, KAMMA H AND OGATA T. (1988). Radiolabelled monoclonal antibody 15 and its fragments for localization and imaging of xenografts of human lung cancer. *J. Natl Cancer Inst.*, **80**, 835-842.

EPSTEIN AL, CHEN F-M AND TAYLOR CR. (1988). A novel method for the detection of necrotic lesions in human cancers. *Cancer Res.*, **48**, 5842-5848.

GOODMAN GE, HELLSTROM I, YELTON DE, MURRAY JL, O'HARA S, MEAKER E, ZEIGLER L, PALAZOLLO P, NICAISE C, USAKEWICZ J AND HELLSTROM KE. (1993). Phase I trial of chimeric (human-mouse) monoclonal antibody L6 in patients with non-small-cell lung, colon, and breast cancer. *Cancer Immunol. Immunother.*, **36**, 267-273.

HAWKINS GA, McCABE RP, KIM C-H, SUBRAMANIAN R, BREDEHORST R, McCULLERS GA, VOGEL C-W, HANNA MG AND POMATO N. (1993). Delivery of radionuclides to pretargeted monoclonal antibodies using dihydrofolate reductase and methotrexate in an affinity system. *Cancer Res.*, **53**, 2368-2373.

HSU S, RAINE L AND FANGER H. (1981). The use of avidin-biotin-peroxidase complex ABC in immunoperoxidase techniques. *J. Histochem. Cytochem.*, **29**, 557.

HUMM JL AND COBB LM. (1990). Nonuniformity of tumor dose in radioimmunotherapy. *J. Nucl. Med.*, **31**, 75-83.

JAIN RK. (1990). Physiological barriers to delivery of monoclonal antibodies and other macromolecules in tumors. *Cancer Res.*, **50**, 814s-819s.

KALOFONOS HP, RUSCHOWSKI M, SIEBECKER DA, SIVOLAPENKO GB, SNOOK D, LAVENDER JP, EPENETOS AA AND HNATOWICH DJ. (1990). Imaging of tumour in pateints with indium-111-labeled biotin and streptavidin-conjugated antibodies: preliminary communication. *J. Nucl. Med.*, **31**, 1791-1796.

LEAR JL, KASLIWAL RK, FEYERABEND AJ, PRATT JP, BUNN PA, DIENHART DG, GONZALEZ R, JOHNSON TK, BLOEDOW DC, MADDOCK SW AND GLENN SD. (1991). Improved tumor imaging with radiolabeled monoclonal antibody with anti-antibody column. *Radiology*, **179**, 509-512.

LEBERTHON B, KHAWLI LA, ALAUDDIN M, MILLER GK, CHARAK BS, MAZUMDER A AND EPSTEIN AL. (1991). Enhanced tumor uptake of macromolecules induced by a novel vasoactive interleukin 2 immunoconjugate. *Cancer Res.*, **51**, 2694-2698.

LE DOUSSAL JM, MARTIN M, GAUTHEROT E, DELAAGE M AND BARBET J. (1989). *In vitro* and *in vivo* targeting of radiolabeled monovalent and divalent haptens with dual specificity monoclonal antibody conjugates: enhanced divalent hapten affinity for cell-bound antibody conjugate. *J. Nucl. Med.*, **30**, 1358-1366.

MARTIN A, PELLEN P, GUITTON C, YOUINOU P, COLLET B, DESRUES B, BOUREL D, DAZORD L AND TOUJAS L. (1989). Characterization of the antigen identified by Po66. *Cancer Immunol. Immunother.*, **29**, 118-124.

NAKAMURA K AND KUBO A. (1994). Effect of interleukin-2 on the biodistribution of technetium-99m-labelled anti-CEA monoclonal antibody in mice bearing human tumour xenografts. *Eur. J. Nucl. Med.*, **21**, 924-929.

PRESS O, EARY JF, APPELBAUM FR, MARTIN PJ, BADGER CC, NELP WB, GLENN S, BUTCHKO G, FISHER D, PORTER B, MATTHEWS DC, FISHER LD AND BERNSTEIN ID. (1993). Radiolabeled-antibody therapy of B-cell lymphoma with autologous bone marrow support. *N. Engl. J. Med.*, **329**, 1219-1224.

REYNOLDS JC, DEL VECCHIO S, SAKAHARA H, LORA ME, CARRASQUILLO JA, NEUMANN RD AND LARSON SM. (1989). Antimurine antibody response to mouse monoclonal antibodies: clinical findings and implications. *Nucl. Med. Biol.*, **16**, 121-125.

ROSENBLUM MG, LAMKI LM, MURRAY JL, CARLO DJ AND GUTTERMAN JU. (1988). Interferon-induced changes in pharmacokinetics and tumor uptake of $^{111}$In-labelled antimelanoma antibody 96.5 in melanoma patients. *J. Natl Cancer Inst.*, **80**, 160-165.

SCHLOM J, EGGENSPERGER D, COLCHER D, MOLINOLO A, HOUCHENS D, MILLER LS, HINKLE G AND SILER K. (1992). Therapeutic advantage of high-affinity anticarcinoma radioimmunoconjugates. *Cancer Res.*, **52**, 1067-1072.

SHARKEY RM, MOTTA-HENNESSY C, PAWLYK D, SIEGEL JA AND GOLDENBERG DM. (1990). Biodistribution and radiation dose estimates for yttrium- and iodine-labelled monoclonal antibody IgG and fragments in nude mice bearing human colonic tumor xenografts. *Cancer Res.*, **50**, 2330-2336.

STICKNEY DR, GRIDLEY DS, KIRK GA AND SLATER JM. (1987). Enhancement of monoclonal antibody binding to melanoma with single dose radiation or hyperthermia. *NCI Monographs*, **3**, 47-52.

WEINSTEIN JN AND VAN OSDOL W. (1992). Early intervention in cancer using monoclonal antibodies and other biological ligands: micropharmacology and the 'binding site barrier'. *Cancer Res.*, **52**, 2747s-2751s.

WELT S, MATTES MJ, GRANDO R, THOMSOM TM, LEONARD RW, ZANZONICO PB, BIGLER RE, YEH S, OETTGEN HF AND OLD LJ. (1987). Monoclonal antibody to an intracellular antigen images human melanoma transplants in *nu/nu* mice. *Proc. Natl Acad. Sci. USA*, **84**, 4200-4204.

ZALCBERG JR, THOMPSON CH, LICHTENSTEIN M AND McKENSIE IF. (1981). Tumor immunotherapy in the mouse with the use of $^{131}$I-labeled monoclonal antibodies. *J. Natl Cancer Inst.*, **72**, 697-704.